# Scar assessment by cardiac MRI can predict outcome in high-risk patients undergoing coronary artery bypass graft (CABG)

**DOI:** 10.1186/1532-429X-13-S1-O88

**Published:** 2011-02-02

**Authors:** Abdalla Elagha, Krishna Kancharla, Peter C Hill, Steven Boyce, Gaby Weissman, Anthon R Fuisz

**Affiliations:** 1 NIH, Bethesda, MD, USA; 2 Washington Hospital Center, Washington DC, DC, USA

## Objectives

The aim of the study is to determine whether the presence and distribution of myocardial infarct scar, detected by delayed-hyperenhancement (DHE-CMR), predicts survival outcome in high-risk patients undergoing CABG.

## Background

Cardiac Magnetic Resonance (CMR)-imaging has an excellent sensitivity and specificity in myocardial scar detection. For patients undergoing CABG, the Society of Thoracic Surgeons (STS) risk-score is an established prognostic tool of survival outcome. The relation between presence of myocardial scar and overall survival after CABG is not well identified. Surprisingly, many patients without evident scar may have unfavorable outcome after CABG, a phenomenon which could be attributed to presence of concomitant non-ischemic cardiomyopathy.

## Methods

We studied a total of 202 patients, who underwent CMR ≤30 days prior to CABG-surgery (2003-2010). The mean and median STS-score were calculated for further risk stratification (higher or lower-risk group). Images of DHE-CMR were acquired in all patients, and classified as (1) No scar (2) Single-vessel territory scar (LAD, LCX, RCA) (3) Multi-vessel territory scars with and without LAD territory. The LVEF, demographic data, risk factors were recorded. Odds ratio, Fisher’s exact, and Kaplan-Meier survival tests were used for statistical analysis.

## Results

**Figure 1:**
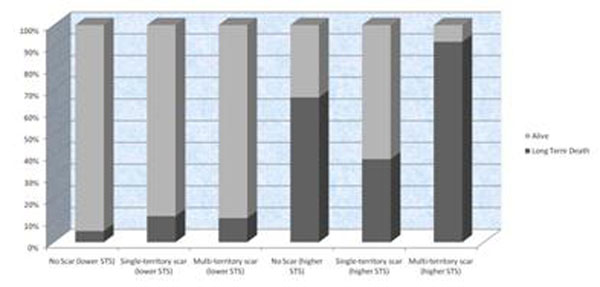


Average follow-up period was 3.6 years. The mean and median STS-score were 1.35% and 2.34%, respectively. Higher and lower risk groups were defined as having STS-score > and ≤ 2.34%, respectively. In patients with lower STS-score, no statistically significant survival difference was observed among different scar distribution groups. However, In patients with higher STS-score, those with multi-segment scars pattern including LAD territory had the highest observed long-term mortality 92% (12 out-of 13), which was significantly higher than both single-territory group 38% (13 out-of 34, p=0.001) and no scar group 67% (8 out-of 12, p=0.003). An interesting observation was noted in higher STS-score group; patients in no scar group had a worse long-term mortality outcome (67%) after CABG compared to 24% (4 out-of 13) in LAD-scar only group (similar EF also), Odds ratio is 6.5, P= 0.02. Figure 1.

## Conclusions

CMR imaging can predict survival in patients undergoing CABG. Scar evaluation by DHE-CMR may have an additive long-term prognostic value to STS-score. In patients with higher STS-score; (1) Those with multi-segment scar pattern have the worst outcome, (2) Some patients without myocardial scar appear to convey an unfavorable prognosis, which represent a vulnerable group that needs further careful evaluation, and should draws attention to exclude concomitant non-ischemic cardiomyopathy.

